# Development and clinical application of radiomics in lung cancer

**DOI:** 10.1186/s13014-017-0885-x

**Published:** 2017-09-15

**Authors:** Bojiang Chen, Rui Zhang, Yuncui Gan, Lan Yang, Weimin Li

**Affiliations:** Department of Respiratory and Critical Care Medicine, West China Hospital of Sichuan University, No. 37, Guo Xue Xiang, Chengdu, Sichuan 610041 China

**Keywords:** Radiomics, Pulmonary nodule, Lung cancer, Phenotype

## Abstract

Since the discovery of X-rays at the end of the 19^th^ century, medical imageology has progressed for 100 years, and medical imaging has become an important auxiliary tool for clinical diagnosis. With the launch of the human genome project (HGP) and the development of various high-throughput detection techniques, disease exploration in the post-genome era has extended beyond investigations of structural changes to in-depth analyses of molecular abnormalities in tissues, organs and cells, on the basis of gene expression and epigenetics. These techniques have given rise to genomics, proteomics, metabolomics and other systems biology subspecialties, including radiogenomics. Radiogenomics is an important revolution in the traditional visually identifiable imaging technology and constitutes a new branch, radiomics. Radiomics is aimed at extracting quantitative imaging features automatically and developing models to predict lesion phenotypes in a non-invasive manner. Here, we summarize the advent and development of radiomics, the basic process and challenges in clinical practice, with a focus on applications in pulmonary nodule evaluations, including diagnostics, pathological and molecular classifications, treatment response assessments and prognostic predictions, especially in radiotherapy.

## Introduction

The suffix “-omics” is now widely used in basic and clinical medical fields to denote the concept of detecting a large dataset and extracting valuable information. It is well known that tumors arise from genetic abnormalities [1]. Treatment responses vary among patients, even those with the same kind of tumor, because of the different patterns of genetic alterations [2, 3]. In 2003, at the annual conference of the European Society for Radiotherapy and Oncology (ESTRO), Baumann et al. proposed the GENEPI project, with the aim of conducting a quantitative study of the relationship between tumor gene expression and radiosensitivity [4]. This project was considered to give rise to the original concept of radiogenomics. The initial definition of radiogenomics was confined to predicting the sensitivity of radiotherapy on the basis of gene expression. Inspired by this vision, many researchers began to analyze the correlation between the gene expression profile and the lesion image, thus expanding the meaning of radiogenomics [5, 6].

In 2012, the sequencing results of renal carcinoma were reported. Researchers found that the tumor gene sequences and their expression levels significantly differed among diverse renal cancer patients and even within the subregions of individual tumor samples. Moreover, phylogenetic reconstruction has revealed branched evolutionary tumor growth, wherein nearly 70% of all somatic mutations are undetectable across the tumor regions [7]. These studies opened the door for explorations of the spatiotemporal heterogeneity of tumors, at both the microscopic and macroscopic levels. Heterogeneity was then confirmed by a subsequent series of studies [8–10]. It has also been observed that tumors with greater genomic heterogeneity are less sensitive to treatment and more likely to metastasize [11–13]. Therefore, heterogeneity evaluations are important for tumor management. However, the value of the traditional small biopsy, even surgical biopsy, is limited, because despite complete resection of tumor tissues with surgery, pathological examinations usually focus on a fraction of the tumor, and the results might not comprehensively reflect the characteristics of the entire tumor. However, spatiotemporal heterogeneity provides a great opportunity for the development of medical imaging technology, which is non-invasive and can be used for continuous and repeated examinations of the entire lesion.

## The advent and development of radiomics

Traditional imaging approaches, such as X-ray radiography, computed tomography (CT), magnetic resonance imaging (MRI) and positron emission tomography (PET), allow extractions of the two-dimensional anatomical and morphological features of tumors semi-quantitatively. However, these methods are incapable of predicting tumor heterogeneity. Thus, there is a pressing need to develop more systematic and comprehensive image technologies. In fact, in addition to displaying conventional morphological signs distinctly, CT and MRI provide a variety of digital pathophysiological details, such as genetic variations and cell functions, thus facilitating individualized selection of therapies. In 2012, a Dutch researcher, Lambin P, proposed the concept of “Radiomics” for the first time and defined it as follows: The extraction of a large number of image features from radiation images with a high-throughput approach [14]. Radiomics has attracted a large amount of attention, and the definition was updated in 2014 to the high-throughput automated (or semi-automated) extraction of large amounts of quantifiable information (or image features) from a region of interest (ROI) in radiographic images. Radiomics was designed to decode the intrinsic heterogeneity, genetic characteristics and other phenotypes of a lesion to improve management [15]. Clearly, radiomics is a product of digital imaging combined with several types of advanced techniques. Radiologists, medical experts, mathematicians and computer scientists are all necessary in this interdisciplinary framework.

## Key technologies and challenges in radiomics

According to the Quantitative Imaging Network (QIN) guidelines established by the National Cancer Institute (NCI), the key technologies and implementation steps of radiomics include the acquisition and reconstruction of standardized images, lesion segmentation, feature extraction, and quantitative data analyses [16].

### Acquisition and reconstruction of standardized images

Original images of radiomics can be derived from anatomical or molecular imaging scans, including CT, MRI and PET. To allow for comparisons and confirmation, the parameters of the original images should be as uniform as possible. However, there are substantial practical challenges.

## CT

CT is the most common imaging modality for radiomics analyses and is allows for easy comparisons across institutions. The imaging performance of CT scanning depends on the imaging technique, scanning parameters and the breathing of the subject [17–19]. The generally accepted evaluation criteria for CT phantoms have been summarized by the American Association of Physicists in Medicine (AAPM) task group reports and comprise four components: slice thickness, Hounsfield unit (HU) variations with electron density, low/high contrast detectability and a region of uniform medium to examine HU changes [16, 20]. The slice thickness, photon statistics and tube voltage are interdependent. Moreover, HUs vary with the reconstruction algorithms and pitches. One lesion scanned using two different reconstruction algorithms or pitches will show significantly different textures [18]. Therefore, efforts must be made to match the original parameters and reconstruction protocols between scanners. Fortunately, the algorithm variations among different vendors allow for acceptable quantitative comparisons, thus providing opportunities for further analyses.

## MRI

In contrast to CT, which reflects the densities of tissues, the signal intensities of MRI are produced by a complex interplay of relaxation times and other acquisition parameters. It is difficult to derive the physical properties of tissues from MRI directly [21]. Certain techniques, such as dynamic contrast-enhanced MRI (DCE-MRI) and diffusion weighted imaging (DWI), have been developed to facilitate the physiological properties assessment. DCE-MRI provides novel insights into the microvasculature, vessel permeability and volume fractions and shows promise as a single tool for tumor volume and contrast enhancement pattern analyses. The results of DCE-MRI depend on the administration method, pulse sequence, contrast agent dose and analysis method. The different parameters used by different investigators act as barriers to comparisons. DWI detects the random motion of water molecules in the body. Unlike water molecules outside the body in random Brownian motion, water molecules in biological tissues engage in restricted movements due to the cell membranes and macromolecular interactions. DWI is primarily used to evaluate acute cerebral infarction, but it has also been used to evaluate tumors. The techniques for DWI acquisitions vary considerably without standardization, and this variability is the greatest challenge to widespread adoption of DWI for tumor assessments [22]. Recently, the Quantitative Imaging Biomarker Alliance (QIBA), also known as the Radiological Society of North America (RSNA), has initiated an effort to standardize the MRI protocol [16].

## PET

PET is a functional imaging modality that uses a tracer known as 18F–fluorodeoxyglucose (18F–FDG). Comparisons of PET images highly depend on the dose of the tracer, the reconstruction of the volume of interest (VOI), and the adherence of the patient to the strict protocol. All of these factors affect the results and are challenges in radiomics analyses [23–25]. Before quantitative image collection, scanner calibration and the scanning protocol need to be uniform to gain comparable results in the standardized uptake value (SUV) of 18-FDG. Additionally, patients are also required to perform specific procedures, including fasting for 4 h to maintain a proper blood glucose level, avoiding high-intensity exercise, and complying with breathing instructions during the examination process. The Society of Nuclear Medicine and the European Association of Nuclear Medicine are working on proposing strict protocols for quantitative PET imaging.

### Lesion segmentation

Lesion segmentation is crucial in radiomics. Although manual segmentation by experienced experts is often regarded as the “gold standard”, the weakness of this labor-intensive step limits the wide use of lesion segmentation in large datasets. In addition, the inevitably high inter-operator variability further makes this technique less feasible. An ideal segmentation method should have four basic features: automation, accuracy, reproducibility and consistency. Recently, a number of automatic or semi-automatic segmentation methods, such as segmentation based on volume CT and an active contour model (ACM), have been developed. However, there is also considerable variation in the results from the same method with different initialization settings. Moreover, different imaging algorithms (CT, MRI or PET) and different anatomical regions (e.g., lung, brain, and liver) have specific requirements. In recent decades, the most commonly used segmentation methods, namely, level set methods, graph cut methods, region growing methods, active contour algorithms and semi-automatic segmentations, have been developed, each with different merits and drawbacks [16]. However, there is no universal segmentation method suitable for all types of medical images. Even when the same algorithm is performed repeatedly with different initializations, the results might be variable. Hence, it is important to develop agreed-upon metrics to evaluate segmentation algorithms. Here, we discuss some major challenges in lung nodule segmentation.

To obtain sufficient information with high fidelity, the slice thickness of the chest CT scan should be 1.5 mm or less [26], and the slice number for each patient might be over 300. Therefore, it is extremely necessary to have an automatic or semi-automatic and reproducible segmentation algorithm, as discussed above. With a strong contrast agent, most of the early stage lung cancers present with homogenously high intensities. The deep contrast with the low-intensity background allows for precise segmentation. However, in cases of ground glass nodules (GGNs), especially the pure GGN without a solid component, the blur differentiation between the lesions and adjacent normal lung tissues makes it difficult to perform a reproducible automatic segmentation. Another common circumstance is the high-intensity tumor is attached to the pleural wall or mediastinum, thus causing automatic segmentations to often fail by over-extending the lesion boundaries. Manual segmentation by radiologists, often called the “gold standard” or “ground truth”, is conventionally subject to overestimation of the lesion volume to ensure covering the entire lesion and exhibits poor reproducibility. Thus, the “gold standard” is not truly accurate. Reproducibility and consistency are usually given priority over accuracy. In other words, a good algorithm should provide reproducible, operator-independent segmentation results automatically. Investigations are continuously being performed in this field [16]. Recently, a semi-automatic segmentation method using three-dimensional lung CT slicers and the GrowCut algorithm has been reported to be able to decrease inter-observer variability and delineation uncertainty. GrowCut is an interactive region segmentation strategy. Before automatic segmentation, a set of labeled pixels for the algorithm should be noted by the user. Then, the algorithm will automatically generate the ROI for the convex hull of the user-labeled pixels and an additional margin. The neighboring pixel weights with a similarity score are used to perform pixel labeling. Pixels with very different weights from those of the neighboring pixels will not be labeled. The foreground and background regions are segmented on the basis of the ROI. Finally, if needed, the ROI for the nodule can be edited manually during a finalization phase [27].

### Feature extraction

Imaging features, including the lesion shape, intensity, texture and wavelet, together with location, can be extracted after lesion segmentation. Feature extraction is also needed for reproducibility, and the information must be informative but not redundant.

## Shape

Segmented lesions are reconstructed into three-dimensional images for further geometric shape descriptions. The maximal and minimal three-dimensional diameters and the total volume are the most commonly used parameters. Similarly to the ratio of the maximal and minimal three-dimensional diameters, the surface-to-volume ratio is also usually used to determine whether the lesion is round or speculated, wherein a round lesion has a much lower value than a speculated lesion with the same volume. Lesion compactness is also calculated. However, the shape is not a specific indicator.

## Intensity

The intensity reflects the lesion voxel value for the selected fractional volume, which is often displayed as the intensity histogram. For CT images, the intensity represents HUs, whereas for PET scans, the intensity indicates the SUVs of FDG. Distribution arguments, including range, mean, median, minimal, maximal, skewness and kurtosis, can be calculated from the intensity histogram to predict the nature of the lesion. More details, such as the percentiles over a set point, can also be determined in medical pattern recognition tasks, but more evidence is needed.

## Texture

The intratumoral texture, introduced by Haralick in 1973, has been widely used to evaluate intertumoral heterogeneity and has potential for enabling differentiation between cancerous and noncancerous lesions [28]. Texture describes the interrelationship between voxels and similar (or dissimilar) contrast values [29]. There are numerous approaches for texture extraction. The most commonly used ones include second-order statistics and co-occurrence matrix features, and the former is preferable. Hundreds of variables are generated, some of which may be redundant. Therefore, it is necessary to evaluate these data by using co-variance [16].

## Wavelet

Wavelets are filter transforms that are determined from a matrix of complex linear or radial “waves” multiplied by the original images. Wavelet features are the transformed domain results representing the intensity and textural information. The most common way to obtain a wavelet is the Coiflet wavelet transformation [30], which is computed on different wavelet decompositions of the original image. Wavelets can be used to extract increasingly coarse texture patterns [31]. Wavelets expressed as ‘Grey Level Nonuniformity HLH’ have been used to describe the intratumoral heterogeneity of lung cancer [32]. However, a standard approach is required for consistency across organizations.

### Quantitative data analyses

In the analyses above, large amounts of data are accessed. It is necessary to establish a standardized database and construct internet sharing platforms with the Internet Cloud technology. This is the foundation for standardized data processing, analyses, and sharing within or across institutions [16, 33]. Furthermore, not all extracted features are useful for a particular task, and the auxiliary information will decrease the analytic power. Therefore, selecting useful information purposefully is crucial for a good radiomics performance. Principal component analyses, machine learning algorithms and statistical approaches are frequently used methods to obtain a new set of features from original information before evaluation of their predictive ability. Then, highly reproducible features with a cutoff value of 0.85 as a concordance correlation coefficient are selected, and this is followed by dynamic range analyses. Although low-dynamic ranging features may be informative, the features with high dynamic ranges are regarded as advantageous. Finally, the remaining redundant features should be removed if the correlation coefficients exceed 0.95. All selected features, characterized as being reproducible, informative and non-redundant, are used to develop classifier models based on machine learning algorithms [16]. The identified models must be inextricably link the imaging, molecular data and clinical data, thus posing many challenges.

## Clinical application of Radiomics in the precision diagnosis and treatment of lung cancer

Because radiomics is a new technology, clinical applications are seldom available. Inspired by radiogenomics, Lambin P has predicted that radiomics will be a powerful tool for indicating tumor genetic heterogeneity, molecular phenotype, pathological diagnosis and clinical prognosis. These factors are highly informative for making clinical decisions [14]. Lung cancer, representing the highest incidence of malignant tumors worldwide, is of perennial interest to researchers. In the subsequent section, we will highlight some recent findings in lung cancer to demonstrate the potential role of radiomics. The first comprehensive application of radiomics in lung cancer was reported by Aerts H in 2014. In that study, 1019 cancer patients, 788 with non-small-cell lung cancers and the other 231 with head-and-neck cancers, were enrolled and divided into seven cohorts for training and validation. Four hundred forty radiomics features quantifying tumor intensity, shape, texture and wavelets were extracted. Along with clinical information and gene expression data, a radiomics heat map was developed to show the clusters of patients with similar radiomics expression patterns. The overall survival prognostic radiomics signature was built on the basis of the first cohort with 422 lung cancers, and its desirable performance was confirmed in the separate lung cancer and head-and-neck cancer groups. Moreover, gene-set enrichment analysis (GSEA) based on another 89 lung cancer cases indicated a satisfactory correspondence between the radiomics features and tumor gene expression data. These data suggested that radiomics is capable of identifying the general tumor prognostic phenotype in lung and head-and-neck cancers from a single-timepoint CT scan. Thus, radiomics may provide an unparalleled opportunity for the wide use of CT imaging in cancer fields, providing the advantages of non-invasiveness and full view. However, the differential value of radiomics in benign and malignant pulmonary nodules was not analyzed in that study [32].

### Differentiating lung cancer from benign pulmonary nodules

In another study, 583 radiomics features from 127 pretreatment pulmonary nodules were extracted to measure the nodule shape, intensity, heterogeneity and other information in multiple frequencies. Patients were divided into 10 subsets randomly with equal sized benign or malignant lesions. A diagnostic model was then executed with the random forest method. Satisfactorily, the sensitivity, specificity and accuracy of this radiomics classifier toward distinguishing malignant primary lung nodules from benign ones achieved 80.0%, 85.5% and 82.7%, respectively; however, the sensitivity of the traditional experienced radiologists’ annotations was only 56.9% with the same specificity [34]. Another study has described quantitative analyses of low-dose CT lung cancer screening images from the well-known National Lung Screening Trial (NLST) at baseline to evaluate whether radiomics could predict the subsequent emergence of cancer. There were two cohorts: one comprised 104 cases and 92 patients with screening-detected lung cancers, and the other comprised a matched group of 208 cases and 196 patients with screening-detected benign pulmonary nodules. Twenty-three stable radiomics features selected by the random forest method predicted nodules that would become cancerous in one or 2 years, with accuracies of 80% (Area Under the Curve, AUC 0.83) and 79% (AUC 0.75), respectively; these results were similar to the accuracy of the McWilliams risk assessment model and exceeded the accuracies of the Lung-RADS and tumor volume approaches [35]. With the popularization of high-resolution CT scanning, subsolid pulmonary nodules are becoming increasingly common. Nearly 34% of persistent subsolid nodules found in the lung cancer screening baseline are diagnosed as malignant in the follow-up screen, a percentage much higher than that for solid lesions (7%) [36]. Therefore, it will be highly beneficial to predict whether the new subsolid nodules are persistent or transient. In a retrospective study of 46 patients with 47 persistent subsolid nodules, and 31 individuals with 39 transient lesions revealed that, beyond the significant differences in age, sex, smoking history and eosinophil counts in the two groups, the CT features of nodule diameter, solid portion size and lesion multiplicity have been found to vary greatly. In terms of radiomics characteristics, the higher mean attenuation, the lower skewness and the ratio of mean attenuation are more inclined to persistent subsolid nodules. Radiomics texture analysis (mean attenuation, skewness and 5 percentile CT number), combined with clinical (eosinophilia) and CT features (lesion size and multiplicity: solitary or multiple), is dramatically more powerful in differentiating persistent pulmonary subsolid nodules from transient ones than the clinical and CT features alone [37]. Textural features with kurtosis analysis have also been found to successfully differentiate pulmonary pre-invasive lesions from invasive lung adenocarcinomas [38]. We believe that radiomics analysis will improve the routine lung cancer diagnosis and support the clinical decision at a low cost.

### Precise pathological and molecular classification in lung cancer

Accurate pathological classification is crucial to planning treatments after a nodule is defined as malignant. Aerts H et al. have observed that 53 radiomics features are significantly associated with lung cancer histology. Moreover, radiomics-based multivariate classifiers, namely, Wavelet_HLL_rlgl_low Gray Level Run Emphasis, Wavelet_HHL_stats_median, Wavelet_HLL_stats_skewness, and Wavelet_HLH_glcm_clus Shade, have been independently validated for the prediction of histological subtypes, even though they achieve a slightly lower predictive accuracy than naive Bayes classifier [39]. With the development of targeted therapies, gene detection and histopathological classification of lung cancer has been recommended as a standard approach by several international authoritative guidelines [40]. However, it is difficult to obtain biopsy tissues in some circumstances because of the inaccessible location of the tumor or the invasive nature of the procedures,. In an Asian cohort of 298 surgically resected peripheral lung adenocarcinomas, 59 of 219 extracted quantitative three-dimensional features have been found to be independent of the epidermal growth factor receptor (*EGFR*) mutation status. Finally, five radiomics features classified into three broad groups have been identified as powerful predictors of the *EGFR* mutation: CT attenuation energy, tumor main direction, and texture, as defined according to wavelets and Laws [41]. Another quantitative CT-based texture analysis has been applied to 48 early-stage non-small-cell lung cancer (NSCLC) patients, and the results have revealed that positive skewness and lower kurtosis are significantly associated with the presence of a *K-ras* mutation. A recursive decision tree with five nodes has been found to improve the differentiation of the *K-ras* mutant from the pan-wild-type NSCLC tumors, with an accuracy of 89.6% [42]. Regarding the relatively rare events of the *ALK* (anaplastic lymphoma kinase), *ROS*1 (c-ros oncogene 1) and *RET* (rearranged during transfection) fusions in lung adenocarcinomas, lower values for kurtosis and inverse variance on three-voxel distance on CT or PET imaging, combined with clinicoradiologic features, such as age, tumor mass and stage, have enabled good discrimination between fusion-positive and fusion-negative tumors, even though the radiomics features of the *ALK* fusion-positive tumors are significantly different from those of the *ROS1/RET* fusion-containing tumors [43].

### Treatment response and prognostic indication in lung cancer, especially in radiotherapy

Another challenge in lung cancer is the difficulty in predicting the treatment response or prognosis. Gratifyingly, in addition to the reports by Aerts H discussed above, several studies have successfully developed radiomics prognostic classifiers for lung cancer patients treated with surgery, radiotherapy or targeted therapies. Eleven stable radiomics feature clusters extracted from the pretreatment CT images with lung cancer have been found to indicate a strong association with prognosis [44]. Local recurrence and distant metastasis are important prognostic factors in cancer patients. Therefore, developing efficient biomarkers to predict patients at high risk of local recurrence or distant metastasis may help to avoid intensive systemic therapy in these subgroups. Coraller TP et al. have constructed a radiomics model with 635 features. Thirty-five have been found to be predictive of metastasis, and 12 have been found to be predictive of survival. The predictive power of the radiomics signature, especially with clinical characteristics, is much higher than the predictive power of the conventional tumor volume [45]. Further neoadjuvant chemoradiation response analysis in 127 locally advanced NSCLC patients has shown that seven radiomics features are predictive of pathologic gross residual disease, and one feature is predictive of a pathologic complete response. Tumors with rounder shapes and heterogeneous textures are more likely to have a poor response to neoadjuvant chemoradiation. However, no conventional imaging features have been found to be predictive [46].

Stereotactic ablative radiotherapy (SABR) is widely applied in lung cancer treatment. However, a benign fibrotic change per imaging has a similar appearance to that of a tumor recurrence, thus representing a challenge for a response assessment after SABR. In a cohort of 45 early-stage lung cancer patients treated with SABR, two regions of interest on the follow-up CT images, a consolidative region and surrounding peri-consolidative region, have been generated semi-automatically and analyzed to predict local recurrence or benign injury within 6 months post-SABR. A radiomics signature consisting of five CT image-appearance features has demonstrated an AUC for local recurrence prediction of 0.85, with an error of 23.7%, false positive rate of 24.0%, and false negative rate of 23.1%. Simultaneously, the prediction efficiency of radiation oncologists or radiologists was found to be much lower, with an error of more than 31% and false negative rate of nearly 99%. These findings suggest that radiomics has the potential to be used as a computer-aided decision tool based on routinely acquired CT imaging. Although this was the first radiomics study to perform recurrence assessments after SABR, further prospective validations using a larger dataset are needed [47]. In subsequent studies, quantitative and CT texture analysis have been applied to quantify radiation-induced lung injury. A higher baseline lung density has been found to be prognostic for radiation-induced lung damage susceptibility [48]. Moreover, compared with the differences in mean density on the CT scan, the combination of mean density changes with the standard deviation dramatically improves the radiation-induced lung damage assessment and has enabled the development of a more accurate predictive mode [49]. A recent study has even shown that the gray-level co-occurrence matrix (GLCM) texture features outperforms the first-order features in distinguishing the lung radiation injury severity levels. A classifier including eight radiomic features has demonstrated a fine dose–response relationship at 3, 6, and 9 months after SBRT [50]. Regarding the overall survival (OS) and recurrence-free survival (RFS) rates for NSCLC patients with SBRT, 24 semantic image features, 219 radiomic features in the baseline planning CT scans and the patient clinical characteristics have been extracted in 92 cases. The Eastern Cooperative Oncology Group (ECOG) performance status, pleural retraction, F2 (short axis × longest diameter) and F186 (Hist-Energy-L1) were included in the model for the two-year OS prediction, and vessel attachment and F2 were included for the RFS prediction. This study has indicated that radiomic features might be helpful in patient stratification and that the features might be powerful in predicting the prognosis of NSCLC patients with SBRT [51]. A similar result has also been reported in another 112 patients. A full analysis of variance has shown that the predictive accuracy depends on feature selection and analysis techniques, thus suggesting that standard methods are required for further investigation [52].

## Conclusions and prospects

The great advantages of radiomics, in the fields of diagnostics and treatment of lung cancer, have been highlighted by numerous studies. Thus, radiomics is expected to be central to precision medicine. The essence of precision medicine is to make personalized decisions for disease prevention, diagnosis and treatment, on the basis of individual patient data gathered through high-precision measurements and efficient information mining and integration. Radiomics can capture detailed information of tumor phenotypes. Full utilization of these cancer-specific characteristics may provide a non-invasive tool for quantifying and monitoring tumors in clinical practice.

Nonetheless, researchers must be aware that in the early stage of development, there are many problems that must be solved in radiomics, from original image extraction to data analysis. Simultaneously, the strong heterogeneity of the tumor presents great challenges to radiomics. Extensive multidisciplinary cooperation is urgently needed to promote the progress of radiomics and produce a revolution in the precision diagnosis and treatment of lung cancer.

## Abbreviations

18F–FDG: 18F–fluorodeoxyglucose
AAPM: American Association of Physicists in Medicine
ACM: Active contour model
ALK: Anaplastic lymphoma kinase
AUC: Area under the curve
CT: Computed tomography
DCE-MRI: Dynamic contrast-enhanced MRI
DWI: Diffusion weighted imaging
ECOG: Eastern Cooperative Oncology Group
EGFR: Epidermal growth factor receptor
ESTRO: European Society for Radiotherapy and Oncology
GGNs: Ground glass nodules
GLCM: Gray level co-occurrence matrix
GSEA: Gene-set enrichment analysis
HGP: Human genome project
HUs: Hounsfield Units
MRI: Magnetic resonance imaging
NCI: National Cancer Institute
NLST: National Lung Screening Trial
NSCLC: Non-small-cell lung cancer
OS: Overall survival
PET: Positron emission tomography
QIBA: Quantitative Imaging Biomarker Alliance
QIN: Quantitative Imaging Network
RET: Rearranged during transfection
RFS: Recurrence-free survival
ROS1: c-ros oncogene 1
RSNA: Radiological Society of North America
SABR: Stereotactic ablative radiotherapy
VOI: Volume of interest
